# Supplementary material for: Tumor Immune Microenvironment Biomarkers for Recurrence Prediction in Locally Advanced Rectal Cancer Patients after Neoadjuvant Chemoradiotherapy
Source: Cancers (Basel). 2024 Sep 30;16(19):3353. doi: 10.3390/cancers16193353 (PMC11475605; doi:10.3390/cancers16193353)
Supplement: Supplementary file 1 [file cancers-16-03353-s001.zip › Supplement Table S1.pdf]

Supplementary Table S1. Total scores of $CD8^+$ T cells, CXCR3 expression and immune phenotypes in tumor specimens (*n*, %).

	CD8+ T cells	CXCR3	Immunophenotype
Grade 1	13 (12)	55 (51)	Immune-desert, 36 (33)
Grade 2	55 (51)	43 (40)	Immune-excluded, 65 (60)
Grade 3	40 (37)	10 (9)	Inflamed, 7 (7)
