# Supplementary figures and images for: Tumor Immune Microenvironment Biomarkers for Recurrence Prediction in Locally Advanced Rectal Cancer Patients after Neoadjuvant Chemoradiotherapy
Source: Cancers (Basel). 2024 Sep 30;16(19):3353. doi: 10.3390/cancers16193353 (PMC11475605; doi:10.3390/cancers16193353)

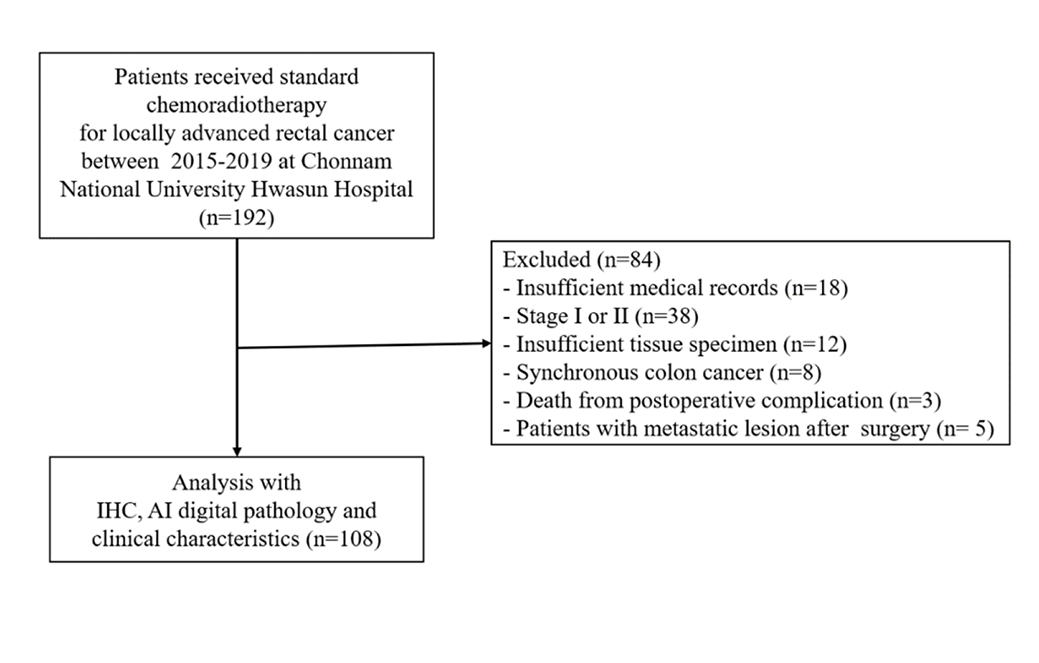

Supplement: Supplementary file 1 [file cancers-16-03353-s001.zip › Supplementary Figure S1.tif]

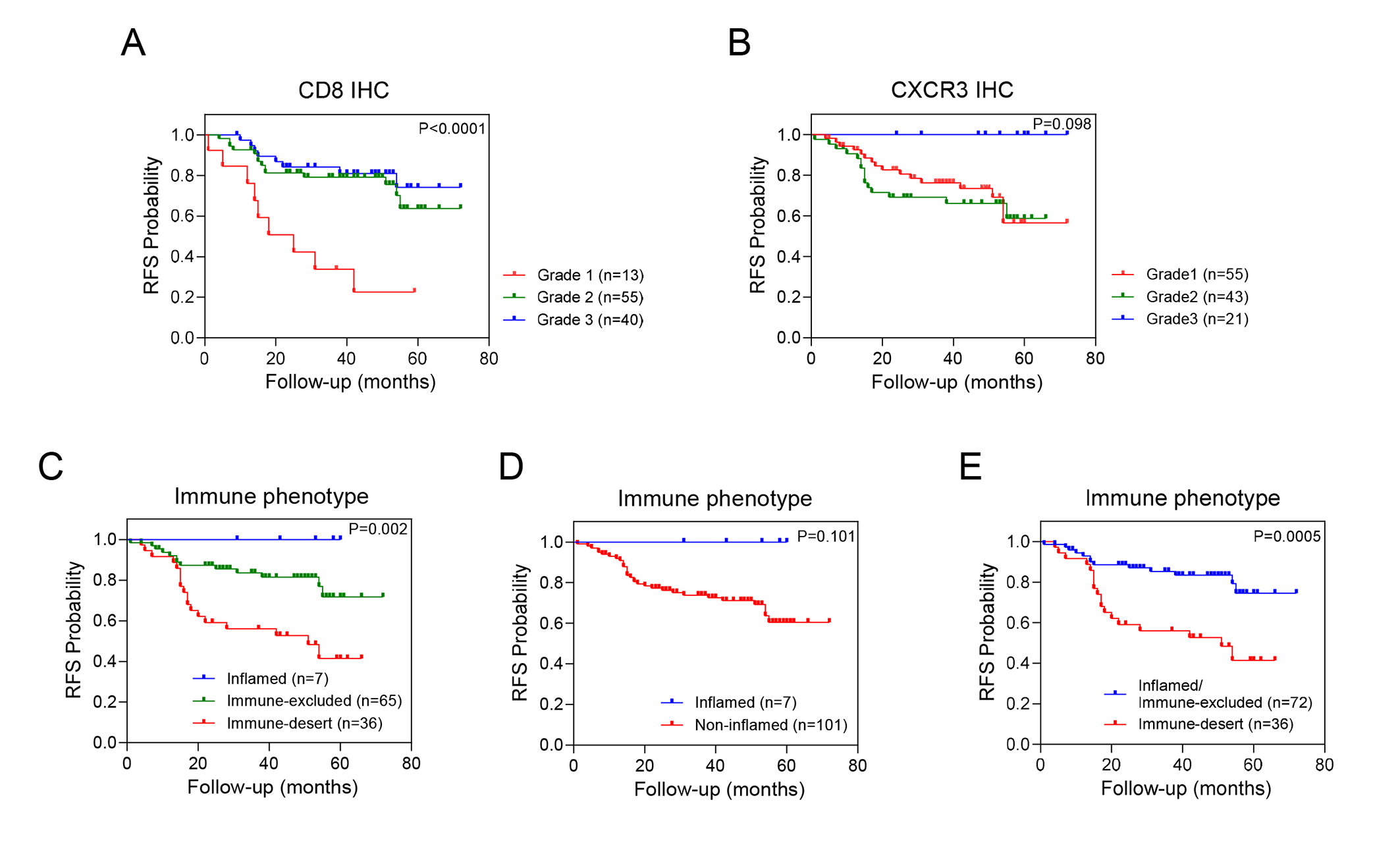

Supplement: Supplementary file 1 [file cancers-16-03353-s001.zip › Supplementary Figure S2.tif]
